# Air pollution during pregnancy and placental adaptation in the levels of global DNA methylation

**DOI:** 10.1371/journal.pone.0199772

**Published:** 2018-07-06

**Authors:** Zhila Maghbooli, Arash Hossein-nezhad, Elham Adabi, Effat Asadollah-pour, Mahsa Sadeghi, Sara Mohammad-nabi, Leila Zakeri Rad, Ali-asghar Malek Hosseini, Mehrnaz Radmehr, Fatemeh Faghihi, Atoosa Aghaei, Abolfazl Omidifar, Yasaman Aghababei, Hadis Behzadi

**Affiliations:** 1 MS Research Center, Neurosciences Institute of Tehran University of Medical Sciences, Tehran, Iran; 2 Department of Medicine, Section of Endocrinology, Nutrition, and Diabetes, Vitamin D, Skin and Bone Research Laboratory, Boston University Medical Center, Boston, Massachusetts, United States of America; 3 Endocrinology and Metabolism Research Institute, Tehran University of Medical Sciences, Tehran, Iran; 4 School of Nursing and Midwifery, Tehran University of Medical sciences, Tehran, Iran; 5 Pregnancy and Gynecology Unit, Milad hospital, Tehran, Iran; 6 Genetic Department, Azad University of Medical Sciences, Tehran, Iran; 7 Department of Medical Laboratory Sciences, School of Allied Medical Sciences, Shahid Beheshti University of Medical Sciences, Tehran, Iran; University of Missouri Columbia, UNITED STATES

## Abstract

**Background:**

Health in early life is crucial for health later in life. Exposure to air pollution during embryonic and early-life development can result in placental epigenetic modification and foetus reprogramming, which can influence disease susceptibility in later life. Objectives: The aim of this paper was to investigate the placental adaptation in the level of global DNA methylation and differential gene expression in the methylation cycle in new-borns exposed to high fine particulate matter in the foetal stage.

**Study design:**

This is a nested case-control study. We enrolled pregnant healthy women attending prenatal care clinics in Tehran, Iran, who were residents of selected polluted and unpolluted regions, before the 14th week of pregnancy. We calculated the regional background levels of particle mass- particles with aerodynamics diameter smaller than 2.5 μm (PM2.5) and 10 μm (PM10)—of two regions of interest. At the time of delivery, placental tissue was taken for gene expression and DNA methylation analyses. We also recorded birth outcomes (the new-born’s sex, birth date, birth weight and length, head and chest circumference, gestational age, Apgar score, and level of neonatal care required).

**Results:**

As regards PM2.5 and PM10 concentrations in different time windows of pregnancy, there were significantly independent positive correlations between PM10 and PM2.5 in the first trimester of all subjects and placental global DNA methylation levels (p-value = 0.01, p-value = 0.03, respectively). The gene expression analysis showed there was significant correlation between S-adenosylmethionine expression and PM2.5 (p = 0.003) and PM10 levels in the first trimester (p = 0.03).

**Conclusion:**

Our data showed prenatal exposures to air pollutants in the first trimester could influence placental adaptation by DNA methylation.

## Introduction

Air pollution is the most pervasive environmental concern and a global public health threat for all people in all age groups. Emerging studies have focused on the exposure effects on early life developmental and child’s health during pregnancy. The effects of exposure to ambient air pollution on prenatal and early childhood health have been systematically reviewed [1]. Most of the evidence has reported the impact of environmental pollution on adverse birth outcomes, such as low birth weight and preterm delivery [1–3].

There is evidence that placental morphology and function are affected by fine particulate matter (PM) in rodents [4]. Moreover, a cohort study suggested that exposure to ambient air pollution during pregnancy reduces the birth size [5]. A recent study has demonstrated the influence of PM on placental transcriptome changes [6]. The authors reported an inverse correlation of placental *BDNF* and *SYN1* gene expression levels at birth with PM2.5 exposure in the first trimester of pregnancy. However, the mechanisms that may underlie the placental adaptations in response to air pollutants are not clear yet. The changes in gene expression associated with placental adaptive responses can be attributed—at least in part—to epigenetic alterations [7, 8]. Compared with other embryonic tissues, placental is more direct contact with air pollution and more susceptible to environmentally induced epigenetic alterations [9].

One of the best-studied epigenetic modifications in the context of altered environment cues is DNA methylation, which involves the addition of a methyl group at the 5’ position of a cytosine adjacent to a guanine (CpG dinucleotide). It seems that maternal exposure to air pollutants is associated with an epigenetic modification, such as DNA methylation in the placenta. Recent genome-wide studies of DNA methylation in human placenta have revealed a number of interesting patterns that are consistent with the proposal that epigenetic regulation may play important roles in the adaptive response to intrinsic and extrinsic factors [10]. Although DNA methylation is essential for imprinted and non-imprinted gene expression that regulate foetus growth and placental function such as nutrition transfer, it is more likely to be vital in the adaptive response of the placenta to environmental signals [11, 12].

The molecular mechanisms that may link various environmental influences with DNA methylation alterations in the placenta and other foetal organs are largely speculated. The activity of placental DNA methyltransferase-1 (*DNMT-1a*)—as a methyl donor group—plays an essential role in the faithful transmission of mother to daughter cells during cell division cycle [13]. *SAMe* is the main substrate involved in methyl group transfers in the methylation cycle.

This study was designed to investigate the placental adaptation in the level of global DNA methylation and differential gene expression of *DNMT-1 α*, and *SAMe*, new-borns exposed to high PM in utero.

## Material and methods

### Study design

This is a nested case-control of a birth cohort study designed by the Tehran University of Medical Sciences. The protocol of the study was published earlier [14]. The research has been supported by the National Institute for Medical Research Development of Iran (Grant No. 940173).

### Study area in Tehran

**Fig 1:**
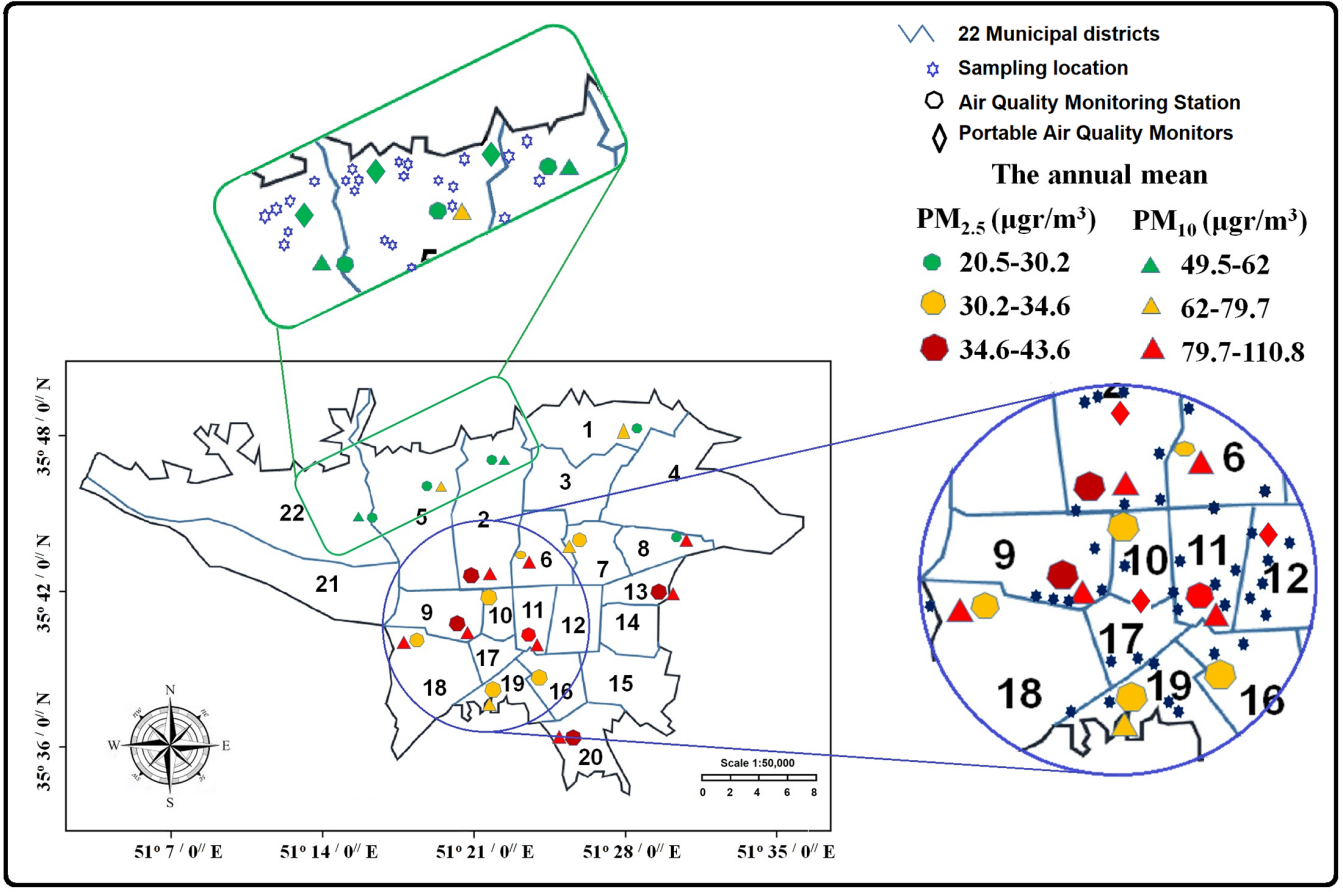
Distribution of monitoring stations and the sampling regions- polluted and non-polluted- in Tehran, Iran. The mean levels of PM2.5 and PM 10 are based on Tehran Air Quality Control Company report in 2015 -http://air.tehran.ir.

The PM concentrations (μg/m3) were detected from 21 monitoring stations of Tehran Air Quality Control Company, to undergo analyses. Based on the annual mean levels of PM2.5 (μg/m3) and PM10 (μg/m3) in 2015, two regions were selected as our study areas; the most polluted (polluted region) and the least polluted (non-polluted region) (http://air.tehran.ir). The most polluted so-called "Traffic Zone" covers the city center during peak traffic hours [15] and the annual mean levels of PM2.5 and PM10 were 34.6–43.6, 79.7–110.8, respectively. The least polluted area is located in northwest of Tehran and the annual mean levels of PM2.5 and PM10 were 20.5–30.2 and 49.5–62, respectively. The detail of distribution of monitoring stations and the sampling regions were summarized in Fig 1.

### Study population

In this study, we enrolled 100 pregnant women who attended prenatal care clinics in two regions in Tehran, Iran: polluted (N = 50) and non- polluted (N = 50) between April 2016 and March 2017. During pregnancy, if a mother moved out of the selected region, was employed, or travelled regularly between polluted and unpolluted regions, she was excluded from the study [14].

Written informed consent was obtained from all study participants in accordance with procedures approved by the Ethical Committee of the National Institute for Medical Research Development (IR.NIMAD.REC.1394.018) of Iran.

The work has been carried out in accordance with the Code of Ethics of the World Medical Association (Declaration of Helsinki) for experiments involving humans, and Uniform Requirements for manuscripts submitted to biomedical journals.

### Exposure measurement

We calculated the regional background daily levels of PM10 and PM2.5 for each mother’s home address from the date of last menstrual period (LMP) to delivery time. The values of air pollutants were obtained from 9 stations of the Tehran Air Quality Control Company in 4×4 km grids [16] in two selected regions. Also, the PM levels we measured manually by using Dylos DC1100 air quality monitors (Dylos Corporation, Riverside, CA, USA) in 3 sites (Fig 1). To explore the potential effect of exposures during pregnancy, we calculated daily regional PM10 and PM2.5 concentrations (micrograms per cubic meter) for each of the three trimesters of pregnancy. We also calculated the mean daily exposure during the whole pregnancy.

Based on WHO air quality guidelines, 10μg/m3 and 20μg/m3 as annual averages of PM2.5 and PM10, respectively, were used to compare the ambient PM with WHO standard values[17]. Also, three interim targets (IT) were used to highlight long-term health effects that relative to the PM concentrations [17].

### Tissue biopsy

The placental tissue biopsy was taken from the foetal side, 1–1.5 cm below the chorioamniotic membrane, at a fixed location in relation to the umbilical cord.

### Global DNA methylation analysis

Genomic DNA was isolated from placental tissue using the standard method. In brief, DNA was extracted by the phenol method from homogenized placental tissues. We determined global DNA methylation, as published earlier [18]. Briefly, RNA was first removed by treating 50 μg DNA in 300 μL 1X Tris-EDTA buffer with RNase A (Fermentas Life Science, Cat No: EN0531) and RNase T1 (Fermentas Life Science, Cat No: EN0541) at final concentrations of 100 μg/mL and 2,000 units/mL, respectively, for 2 h at 37°C, which was followed by ethanol precipitation. The dissolved DNA was then digested with 50 μg/mL DNase I (Fermentas Life Science, Cat No: EN0521) for 14 h at 37 °C, denatured by heating at 100 °C for 3 minutes, and rapidly cooled on ice. Then, 2 volumes 30 mM sodium acetate pH 5.2 (Carlo Erba Reagenti SpA, Cat No: 478167) with ZnSO_4_ and nuclease P1 (Sigma-Aldrich, Cat No: N8630) at final concentrations of 1mM and 50 μg/ml, respectively, were added and the mixture incubated for a further 16 h at 37 °C. Hydrolyzed DNA from all subjects was maintained at -80°C until analysis. Global DNA methylation was expressed as the percentage of 5-methyldeoxycytidine (5-mdC) versus the sum of 5-mdC and deoxycytidine (dC): [5-mdC/(5-mdC + dC)]%.

### Gene expression analysis

We extracted RNA from placental tissues using a Qiagen kit (QIAGEN NV, Venlo, and the Netherlands). The integrity of total RNA was checked by electrophoresis through an agarose gel. After DNase I treatment, 1 μg of total RNA was reverse transcribed using random hexamers as primers and Superscript II reverse transcriptase (Fermentaze). Gene expression was analysed by using real time polymerase chain reaction after complementary DNA synthesis.

Candidate genes include DNA (cytosine-5)-methyltransferase-1-alpha (*DNMT1-a*), *SAMe* and *GAPDH*. Expression of the genes was determined by quantitative real-time polymerase chain reaction using an Applied Biosystems (ABI) 7900HT Real-Time PCR System. The presence of specific gene products was also confirmed with melting curve analysis. The primer sequences were used as follow: DNMT1a (f) 5’ CCT AGC CCC AGG ATT ACA AGG, DNMT1a (r) 5’ ACT CAT CCG ATT TGG CTG CTC TTT C, SAMe (f) 5’ CACCATCAAGCACATCGGCTA, SAMe (R) 5’ CCGAACATCAAACCCTGATCTC, GAPDH (f) 5’ TTC TCT GAT TTG GTC GTA TTG G, GAPDH (r) 5’ CAT GTA AAC CAT GTA GTT GAG GTC.

All samples were amplified in duplicate, and the mean was obtained for further calculations. The expression amounts were calculated by using the expression of the GAPDH, which was used as a reference gene. The Ct for each sample was normalized to the corresponding sample geometric mean of GAPDH.

### Statistical analysis

We presented categorical data as frequencies (%) and numbers, and continuous data as mean ± standard error for variables with normal distribution or median (IQR) for variables without normal distribution. We used chi-square test to compare the prevalence of adverse birth outcomes in the two regions. Mann Whitney U test compared the differences in global DNA methylation levels in placenta of pregnant women in the two regions (polluted and non-polluted).

We used Spearman correlation coefficients to assess the association of global DNA methylation from placental tissue with PM10 and PM2.5 concentrations. A univariate model was used to determine the independent association of PM10, and PM2.5 exposures during pregnancy with placental global DNA methylation.

The 2^(-ΔCt)^ formula was used to calculate relative transcript abundance. Fold changes were used to compare gene expression differences of all included genes in placental tissue between the two groups, that is, pregnant women who lived in polluted and non-polluted regions. We considered a fold-change value>1.5 for gene expression and 2-tailed *P* values <.05 as statistically significant.

## Results

In 2016, the annual mean levels of PM2.5 (μg/m^3^) and PM10 (μg/m^3^) in all regions of Tehran, Iran, were higher than WHO air quality guidelines (AQG), mean (min, max) for PM2.5; 87.29 (33, 156) and for PM10; 60.20 (19, 135).

Apart from the values in the WHO guidelines, three interim targets (IT) were defined [17]. The annual mean levels of PM2.5 was in IT1 category and the interim of PM10 was in IT2 category. Indeed, only 22 days were in clean criteria.

### Study population characteristics and exposure levels

Among the 100 pregnant women enrolled in the study, four women were excluded because they moved out of the selected region during pregnancy and four women delivered in other cities. For the purposes of data analysis, 92 women were included: 48 women who lived in a polluted region and 44 women who lived in a non-polluted region. The baseline characteristics of mothers and their new-borns are shown in Table 1. There were no significant differences in age, pre-gestational BMI, gravity, and parity. The mean regional background levels of PM10, PM2.5 for total pregnancy duration and each of the three trimesters of pregnancy were presented in Table 2.

**Table 1:** Baseline characteristics of the mother-newborn pairs in two regions, mean± SE or number (%).

	Polluted (48)	Non-polluted (44)	p-value
**Maternal**			
Age, year	30.00±0.71	30.09±0.79	0.93
Pre. Gestational BMI, Kg/m^2^	25.31±0.53	24.51±0.60	0.33
Weight. Gain during pregnancy, Kg	13.31±0.70	13.48±1.29	0.78
Systolic Blood Pressure, mmHg	114.00±2.33	108.84±1.51	0.07
Diastolic Blood Pressure, mmHg	73.77±2.16	71.42±1.76	0.40
Gravity	1.9±0.14	2.1±0.13	0.35
Previous Abortion	12 (25)	13 (29)	0.62
Education level			0.15
Middle-school	3 (6)	4 (9)	
High-school	32 (66)	21 (48)	
University	13 (28)	19 (43)	
Social Economical Status			0.83
Land lord	20 (42)	19 (43)	
Tenant	28 (58)	25 (57)	
Taking Acid folic during pregnancy	23 (48)	24	0.69
Taking Iron during pregnancy	38 (79)	34	0.85
Taking perinatal Multivitamin	42 (87.5)	40	0.69
GDM	-	3	0.09
Hypertension during	8 (16)	4	0.36
Social Class			-
Equal	38 (79)	30 (68)	
More save	0	0	
More pay	2 (4)	0	
No response	8(17)	14 (32)	
**Newborn**			
Sex (Female)	24 (50)	18 (41)	0.35
Gestational age, weeks	38.93±0.16	38.48±0.22	0.09
Pre-term (<37 weeks)	0	1 (2)	0.44
Season at birth			0.09
Winter	21 (44)	12 (27)	
Fall	27 (56)	32 (73)	
Apgar score 1min			0.62
9	42 (88)	41 (93)	
8	3 (6)	3 (7)	
<8	3 (6)	0	
Apgar score 5min			0.63
9–10	46 (96)	44 (100)	
<9	2 (4)	0	
Birth weight, gr	3243.25±63.30	3285.25±64.47	0.64
Birth length, cm	50.06±0.38	50.30±0.37	0.67
Head circumference, cm	34.36±0.47	34.73±0.21	0.27
Chest circumference, cm	33.93±0.47	33.30±0.31	0.40

Numerical variables were expressed as the mean ± standard error (SE) and categorical variables were presented as frequencies (%) and numbers. (-) not statistically was tested because of incomplete data.

**Table 2:** Fine particulate matter levels in two regions; polluted and non-polluted.

	Polluted (N = 48)	Non-polluted (N = 44)	P-value
**PM 2.5 (μ/m**^**2**^**)**			
Whole pregnancy	37.12±.50	25.18±.68	0.0001
Trimester 1	30.99±0.86	20.43±.68	0.0001
Trimester 2	38.44±0.71	26.37±1.23	0.0001
Trimester 3	42.44±.74	29.04±1.11	0.0001
**PM 10 (μ/m**^**2**^**)**			
Whole pregnancy	91.45±2.51	70.43±1.13	0.0001
Trimester 1	74.34±2.66	64.97±2.52	0.01
Trimester 2	94.88±3.35	74.06±1.65	0.0001
Trimester 3	104.89±2.61	72.13±1.33	0.0001

Numerical variables were expressed as the mean ± standard error (SE). PM2.5; fine particulate matter with a diameter 2.5 μm, PM 10; fine particulate matter with a diameter 10 μm.

### Fine particulate matter levels and perinatal outcome

Based on PM concentrations, there was no significant correlation between birth outcomes, including gestational age, weight, length, head and chest circumference, at the time of birth and PM2.5 and PM10 levels in whole pregnancy or in each trimester (p-value>0.05) (S1 Table).

### Quantification of placental global DNA methylation related to maternal age, pre-gestational BMI, and gravity and birth outcomes

In all subjects, there were no significant correlations between placental global DNA methylation levels with maternal age, pre-gestational BMI, gravity, and parity (p-value>0.05) (S2 Table). Similar results were found in each region (p-value>0.05).

Regarding birth outcomes, there was no significant correlation between maternal and placental global DNA methylation levels and birth outcomes, including gestational age, weight, length, and head and chest circumference at the time of birth (p-value>0.05) (S3 Table).

### Placental global DNA methylation in polluted region compared with non-polluted region

No significant difference was observed in the global DNA methylation levels of placental in two regions: non-polluted vs. polluted regions (2.44 (0.86) vs. 2.59 (0.70), p-value = 0.42, respectively).

As regards PM2.5 and PM10 concentrations in different time windows of pregnancy, there was significant positive correlations between PM2.5 and PM10 in the first trimester of all subjects in two regions and placental global DNA methylation levels (Table 3). Also, there was significant correlation between PM2.5 in third trimester of subjects in polluted region and placental global DNA methylation levels (r = 0.31, p = 0.04).

**Table 3:** Correlation between fine particulate matter concentrations and placental DNA methylation in different time windows of pregnancy.

	Total		polluted		Non-polluted	
	Placental CM (%)		Placental CM (%)		Placental CM (%)	
	Spearman’s rho	p-value	Spearman’s rho	p-value	Spearman’s rho	p-value
**PM.2.5-** Whole pregnancy	.17	.11	.31*	.02	.14	.41
PM2.5- Trimester 1	.26*	.01	.40**	.003	.42**	.009
PM2.5-Trimester 2	-.005	.96	-.07	.62	.04	.79
PM2.5 –Trimester 3	.08	.43	.29*	.04	-.09	.59
**PM10** -Whole pregnancy	.14	.20	.16	.25	.25	.13
PM10 –Trimester 1	.38**	.0001	.42**	.002	.40*	.01
PM10 –Trimester 2	.10	.34	.10	.48	.27	.11
PM10 –Trimester 3	-.07	.50	-.06	.67	-.18	.29

*correlation is significant at the 0.05 level

**correlation is significant at the 0.01 level

In univariate model, after adjusting for gestational age, new-born sex, and region, there was only significant association between PM2.5 (p-value = 0.03) and PM10 (p-value = 0.01) only in the first trimester and placental global DNA methylation levels.

### Differently expressions of DNMT-1 α, and SAMe genes in placental tissue

**Fig 2:**
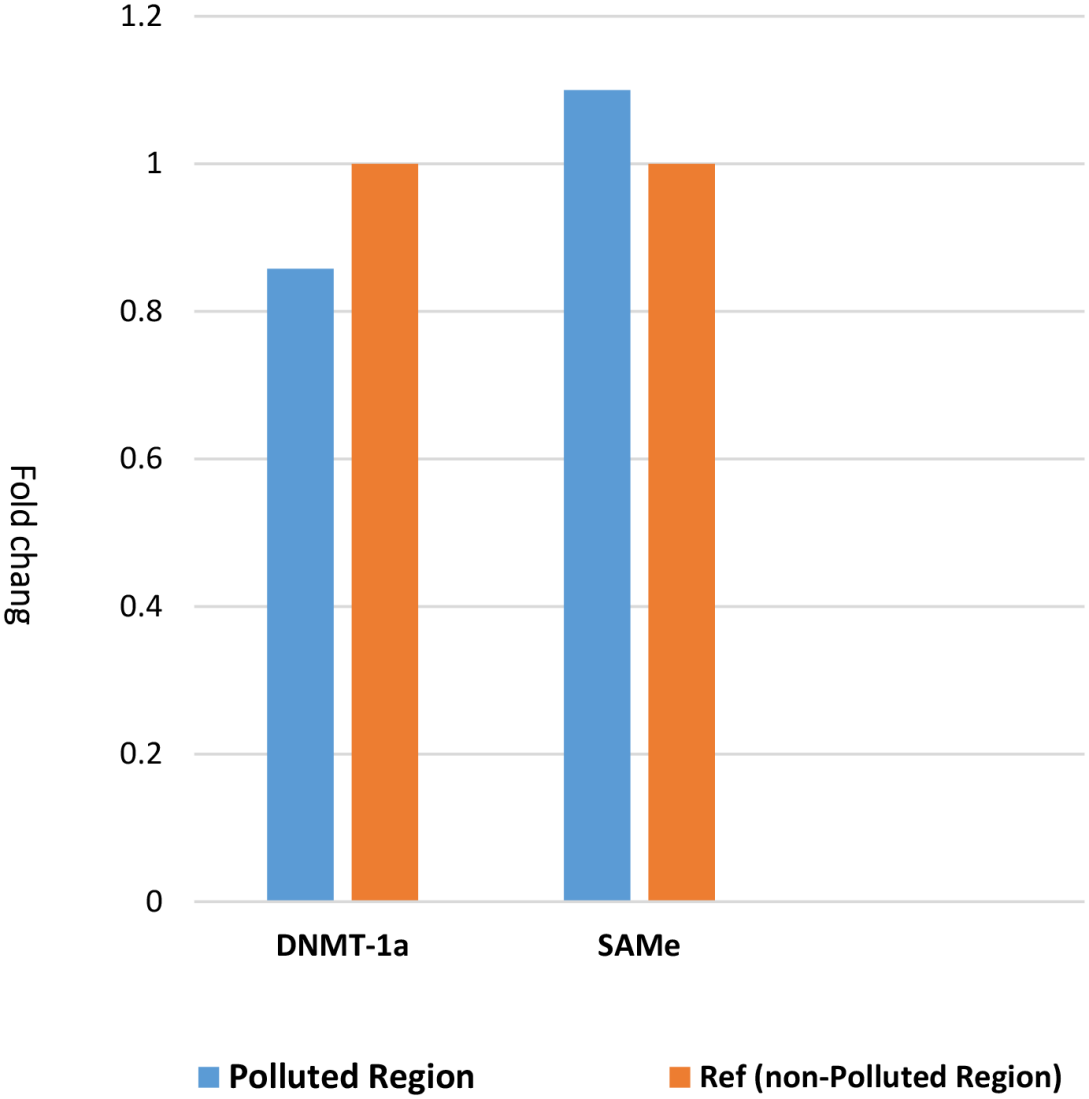
Differently expressed genes in placental tissues of mothers who lived in polluted region compared to those in non-polluted region. Not differentially expressed selected genes in placental samples from mothers in polluted compare to ones in non-polluted regions; with a fold change <1.5 (fold change values: DNMT-1 α = -1.2, and SAMe = 1.1).

Regarding selected regions, the data analysis showed not differentially expressed selected genes in placental samples from mothers in polluted compared to ones in non-polluted regions; with a fold change <1.5 and/or *p* value> 0.05 (fold change values: DNMT-1 α = -1.2, and SAMe = 1.1) (Fig 2).

Based on PM concentrations, there was only significant correlation between *SAMe* expression and PM2.5 in first (r = -0.37, p = 0.01) and third (r = -0.42, p = 0.003) trimesters and PM10 levels in first trimester (r = -0.31, p = 0.04) (Table 4).

**Table 4:** Correlation between fine particulate matter concentrations and RNA expression levels of *DNMT-1 α* and *SAMe*.

		Total		Polluted		Non-polluted	
		*DNMT-1 α*	*SAMe*	*DNMT-1 α*	*SAMe*	*DNMT-1 α*	*SAMe*
PM2.5-Whole pregnancy	rho	-.10	-.02	-.15	-.37*	.02	-.08
	p-value	.38	.86	.31	.01	.91	.63
PM2.5-Trimester 1	rho	-.17	-.06	-.23	-.42**	-.15	-.17
	p-value	.12	.59	.12	.003	.39	.31
PM2.5-Trimester 2	rho	-.04	.06	-.02	.114	.01	-.29
	p-value	.68	.57	.89	.45	.95	.08
PM2.5-Trimester3	rho	-.07	.05	-.13	-.42**	.02	.05
	p-value	.53	.63	.36	.003	.90	.77
PM10-Whole pregnancy	rho	-.04	-.19	-.15	-.27	.02	-.08
	p-value	.71	.09	.29	.06	.92	.63
PM10-Trimester 1	rho	-.09	-.40**	-.120	-.31*	-.15	-.17
	p-value	.42	.0001	.42	.03	.39	.31
PM10-Trimester 1	rho	-.05	-.08	-.19	-.23	.01	-.29
	p-value	.66	.46	.17	.12	.95	.08
PM10-Trimester 1	rho	.007	.084	.08	-.11	.02	.05
	p-value	.95	.45	.57	.46	.90	.77

Rho; Spearman,

*correlation is significant at the 0.05 level

**correlation is significant at the 0.01 level

DNMT1-a; relative gene expression of DNMT1-a, SAMe; relative gene expression of SAMe

In total, our data showed no significant association between RNA expression of *DNMT-1 α* and *SAMe* (r = 0.09, p = 0.42).

### Correlation between placental global DNA methylation levels and RNA expression levels of *DNMT-1 α*, and *SAMe*

There were significant negative correlations between placental global DNA methylation and gene expressions of *SAMe* (r = -0.24, p = 0.039) and *DNMT-1 α*(r = -.26, p = 0.028).

Based on selected regions, there were only significant negative correlation between gene expression levels of *SAMe* and global DNA methylation levels (r = -0.31, p = -0.04) in placental tissues of mothers who lived in a polluted region.

## Discussion

Growing evidence suggests that placental adaptations in the molecular levels are crucial in ‘the developmental programming of adult diseases’ [10]. During pregnancy, developmental adaptations due to epigenetic modification may not only permanently ‘program’ the foetus and cause adverse pregnancy outcomes but can also lead to possible future diseases in adult life. We supposed exposure to fine particulate matter (PM) during early time of development could affect placental DNA methylation levels.

To shed light on the possible impacts of maternal air pollution exposure and to find out important clues relating to exposure, we measured the levels of PM10 and PM2.5 at different times of pregnancy in women who lived in polluted and non-polluted regions of Tehran, Iran.

Our observations in different time windows of pregnancy on PM-induced placental global DNA methylation levels showed significant direct independent correlation between PM2.5 and PM10 concentrations with placental global DNA methylation only in the first trimester. There was no significant difference between placental global DNA methylation levels between mothers who live in polluted and non-polluted regions. Not surprisingly, the first trimester of pregnancy is the critical time for foetal development. As the function of placental is important in environmental challenges and foetal programming, molecular regulations or epigenetic modifications that occur in placental tissue are vital to modify the effect of interactions between environmental factors and foetus.

Recent genome-wide studies of DNA methylation in human placenta have revealed a number of interesting patterns that are consistent with the proposal that epigenetic regulation may play important roles in the adaptive response to intrinsic and extrinsic factors [19, 20]. It appears that alterations in genomic DNA methylation as well as gene-specific DNA methylation and gene expression patterns during foetal development can result from the exposure to fine particulate matter. In line with our findings, both animal models studies and human ones have reported that exposure to environmental pollutants is associated with changes in DNA methylation patterns [21–24].

In our data, there was no significant difference in birth outcomes, including birth weight, length, head and chest circumference of new-borns, in the two regions. Also, we did not find any significant correlation between PM levels and birth outcomes. As a dynamic process, in response to maternal environmental cues, placental epigenetic modifications occur to improve foetal viability until birth. This dynamic placental adaptation is an additional protection layer of the foetus in the deal with abnormal maternal–foetal environment [10]. However, depending on the severity of abnormal maternal environment conditions and timing, not only may the placental adaptive response be insufficient to maintain a normal foetal growth, but also induce detrimental secondary effects, such as changes in the placental vascular resistance and abnormal placental hormone metabolism [10].

Our second aim was to evaluate RNA expression of selected genes contributing to DNA methylation patterns in response to inhalation of particulate matter during pregnancy; *DNMT-1a*, and *SAMe*. Our assessments showed significant mild-negative correlations between placental global DNA methylation and gene expressions of *SAMe* and *DNMT-1a*. A positive correlation between donor methyl and active enzyme and DNA methylation levels would be expected. It can be attributed to placental adaption in the levels of gene expressions independent of global hyper-methylation in response to PM exposure. On the other hand, it is possible that the indirect correlation is a placental plasticity to balance DNA methylation levels in the last two trimesters. Otherwise, other epigenetic mechanisms and intracellular molecular pathways could modify gene expression patterns of placental tissue in response to abnormal extra-uterine condition. As a ‘maintenance’ methyltransferase, *DNMT-1a* functions in accurate propagation of the genomic methylation profile from mother to daughter cells following cell division [25]. Despite this unjustifiable reverse correlation between global hyper-methylation and enzyme activity of *DNMT* and *SAMe*, it is clear that *DNMT-1a* levels are important in some locus-specific or repeat-based DNA methylation and, consequently, in gene expression levels for correct placental development. Alterations of DNA methylation patterns could modulate immune responses and inflammatory genes, in response to the inhalation of particulate matter. Of note, the placenta is a remarkable organ; during the short time of pregnancy, especially in the first trimester, placental enables the foetus to survive within the confines of the abnormal extra- and intra-uterine environment.

Several limitations in our study are worth noting. Firstly, some confounding factors, such as some lifestyle-related factors, environmental tobacco smoke, the season, and environmental temperature, could have a plausible impact on placental tissue DNA methylation. To minimize the effects of lifestyle and regional differences on methylation patterns, we adjusted for the mother’s socioeconomic status, and maternal diet in the analysis. We also considered exposure to other air pollutants, such as second-hand smoke in our analysis. More importantly, mothers who smoke or live with a smoker were excluded from our study. It should be noted that we may ignore some unknown factors that have influences on blood and tissue DNA methylation, as well as levels of air pollutants. Secondly, it is likely that the results due to paired comparisons may not be significant when multiple comparison statistics are considered. This study certainly makes multiple comparisons and it is difficult to ascertain the effect on significance. Therefore, more large-scale studies need to evaluate the role of air pollution components in placental tissue adaptations.

### Conclusion

All things considered, Tehran is a one of the most polluted cities in the world with PM2.5 levels 8.7 times and PM10 levels four times values higher than those recommended by the WHO. Our data highlight the role of maternal environmental exposures during first trimester on placental adaptive responses in the levels of DNA methylation. Hence, the first trimester of pregnancy is likely most relevant to PM exposure and may represent a higher risk and critical time for influencing disease susceptibility in later life. Consequence, in first trimester, prenatal care and classroom-educations of health and air pollution could be helpful in preventing air pollution harmful effects. Undoubtedly, perinatal care during first trimester is important; it is suggested that prevention of air pollution be considered in routine antepartum algorithm in polluted cities.

Though our study shows that exposure to PM during first trimester is associated with placental global DNA methylation levels, it is recommended that further studies to clarify the mechanisms of placental adaptation to prevent the influence of environmental pollutants on the early development of the foetus and disease susceptibility in adult ages.

## Supporting information

S1 Table Relationship between fine particulate matter and birth outcomes. Based on PM concentration, there were not any significant correlation between birth outcome including gestational age, weight, length, and head and chest circumference at the time of birth and PM2.5 and PM10 concentrations in whole pregnancy or each trimester (p>0.05).
(DOCX)

S2 Table Relationship between placental DNA methylation levels and modification factors. In all subjects, there were not any significant correlations between placental global DNA methylation levels with maternal and gestational ages, pre gestational BMI, gravity and parity (p-value>0.05).
(DOCX)

S3 Table Relationship between placental DNA methylation levels and birth outcomes. Regarding to birth outcomes, There were not any significant correlation between placental global DNA methylation and birth outcome including gestational age, weight, length, and head and chest circumference at the time of birth (p>0.05).
(DOCX)

## References

[1] Glinianaia SV, Rankin J, Bell R, Pless-Mulloli T, Howel D. Particulate air pollution and fetal health: a systematic review of the epidemiologic evidence. Epidemiology (Cambridge, Mass). 2004;15(1):36–45. Epub 2004/01/09. 10.1097/01.ede.0000101023.41844.ac 14712145

[2] Schwartz J, Dockery DW, Neas LM, Wypij D, Ware JH, Spengler JD, et al Acute effects of summer air pollution on respiratory symptom reporting in children. American journal of respiratory and critical care medicine. 1994;150(5 Pt 1):1234–42. Epub 1994/11/01. doi: 10.1164/ajrccm.150.5.7952546 . 7952546 10.1164/ajrccm.150.5.7952546

[3] Maisonet M, Correa A, Misra D, Jaakkola JJ. A review of the literature on the effects of ambient air pollution on fetal growth. Environmental research. 2004;95(1):106–15. Epub 2004/04/08. doi: 10.1016/j.envres.2004.01.001 . 15068936 10.1016/j.envres.2004.01.001

[4] Veras MM, Damaceno-Rodrigues NR, Caldini EG, Maciel Ribeiro AA, Mayhew TM, Saldiva PH, et al Particulate urban air pollution affects the functional morphology of mouse placenta. Biology of reproduction. 2008;79(3):578–84. doi: 10.1095/biolreprod.108.069591 . 18509159 10.1095/biolreprod.108.069591

[5] Ballester F, Estarlich M, Iniguez C, Llop S, Ramon R, Esplugues A, et al Air pollution exposure during pregnancy and reduced birth size: a prospective birth cohort study in Valencia, Spain. Environmental health: a global access science source. 2010;9:6 doi: 10.1186/1476-069X-9-6 . 20113501 10.1186/1476-069X-9-6 PMC2845572

[6] Saenen ND, Plusquin M, Bijnens E, Janssen BG, Gyselaers W, Cox B, et al In Utero Fine Particle Air Pollution and Placental Expression of Genes in the Brain-Derived Neurotrophic Factor Signaling Pathway: An ENVIRONAGE Birth Cohort Study. Environmental health perspectives. 2015;123(8):834–40. 25816123 10.1289/ehp.1408549 PMC4529006

[7] Nelissen EC, van Montfoort AP, Dumoulin JC, Evers JL. Epigenetics and the placenta. Human reproduction update. 2011;17(3):397–417. doi: 10.1093/humupd/dmq052 . 20959349 10.1093/humupd/dmq052

[8] Banister CE, Koestler DC, Maccani MA, Padbury JF, Houseman EA, Marsit CJ. Infant growth restriction is associated with distinct patterns of DNA methylation in human placentas. Epigenetics. 2011;6(7):920–7. Epub 2011/07/16. doi: 10.4161/epi.6.7.16079 . 21758004 10.4161/epi.6.7.16079 PMC3154432

[9] Fowden AL, Coan PM, Angiolini E, Burton GJ, Constancia M. Imprinted genes and the epigenetic regulation of placental phenotype. Progress in biophysics and molecular biology. 2011;106(1):281–8. doi: 10.1016/j.pbiomolbio.2010.11.005 . 21108957 10.1016/j.pbiomolbio.2010.11.005

[10] Sandovici I, Hoelle K, Angiolini E, Constancia M. Placental adaptations to the maternal-fetal environment: implications for fetal growth and developmental programming. Reproductive biomedicine online. 2012;25(1):68–89. doi: 10.1016/j.rbmo.2012.03.017 . 22560117 10.1016/j.rbmo.2012.03.017

[11] Fowden AL, Sibley C, Reik W, Constancia M. Imprinted genes, placental development and fetal growth. Hormone research. 2006;65 Suppl 3:50–8. doi: 10.1159/000091506 . 16612114 10.1159/000091506

[12] Koukoura O, Sifakis S, Spandidos DA. DNA methylation in the human placenta and fetal growth (review). Molecular medicine reports. 2012;5(4):883–9. doi: 10.3892/mmr.2012.763 . 22294146 10.3892/mmr.2012.763 PMC3493070

[13] Hochberg Z, Feil R, Constancia M, Fraga M, Junien C, Carel JC, et al Child health, developmental plasticity, and epigenetic programming. Endocrine reviews. 2011;32(2):159–224. doi: 10.1210/er.2009-0039 . 20971919 10.1210/er.2009-0039 PMC3365792

[14] Maghbooli Z, Hossein-Nezhad A, Ramezani M, Moattari S. Epigenetic Alterations and Exposure to Air Pollutants: Protocol for a Birth Cohort Study to Evaluate the Association Between Adverse Birth Outcomes and Global DNA Methylation. JMIR research protocols. 2017;6(2):e29 doi: 10.2196/resprot.7114 . 28232302 10.2196/resprot.7114 PMC5344983

[15] Halek F, Kavousi-rahim A. GIS ASSESSMENT OF THE PM 10, PM 2.5 AND PM 1.0 CONCENTRATIONS IN URBAN AREA OF TEHRAN IN WARM AND COLD SEASONS. The International Archives of Photogrammetry, Remote Sensing and Spatial Information Sciences. 2014;40(2):141.

[16] Seigneur C, Dennis R. Atmospheric modeling Technical Challenges of Multipollutant Air Quality Management: Springer; 2011 p. 299–337.

[17] Krzyzanowski M, Cohen A. Update of WHO air quality guidelines. Air Quality, Atmosphere & Health. 2008;1(1):7–13.

[18] Maghbooli Z, Hossein-nezhad A, Larijani B, Amini M, Keshtkar A. Global DNA methylation as a possible biomarker for diabetic retinopathy. Diabetes/metabolism research and reviews. 2015;31(2):183–9. Epub 2014/07/30. doi: 10.1002/dmrr.2584 . 25069700 10.1002/dmrr.2584

[19] Su J, Wang Y, Xing X, Liu J, Zhang Y. Genome-wide analysis of DNA methylation in bovine placentas. BMC genomics. 2014;15:12 Epub 2014/01/09. doi: 10.1186/1471-2164-15-12 . 24397284 10.1186/1471-2164-15-12 PMC3893433

[20] Keravnou A, Ioannides M, Tsangaras K, Loizides C, Hadjidaniel MD, Papageorgiou EA, et al Whole-genome fetal and maternal DNA methylation analysis using MeDIP-NGS for the identification of differentially methylated regions. Genetics research. 2016;98:e15 Epub 2016/11/12. doi: 10.1017/S0016672316000136 . 27834155 10.1017/S0016672316000136 PMC6865150

[21] Saenen ND, Vrijens K, Janssen BG, Roels HA, Neven KY, Vanden Berghe W, et al Lower Placental Leptin Promoter Methylation in Association with Fine Particulate Matter Air Pollution during Pregnancy and Placental Nitrosative Stress at Birth in the ENVIRONAGE Cohort. Environmental health perspectives. 2016 Epub 2016/09/14. doi: 10.1289/ehp38 . 27623604 10.1289/EHP38 PMC5289914

[22] Kingsley SL, Eliot MN, Whitsel EA, Huang YT, Kelsey KT, Marsit CJ, et al Maternal residential proximity to major roadways, birth weight, and placental DNA methylation. Environment international. 2016;92–93:43–9. Epub 2016/04/09. doi: 10.1016/j.envint.2016.03.020 . 27058926 10.1016/j.envint.2016.03.020 PMC4913202

[23] Wilhelm-Benartzi CS, Houseman EA, Maccani MA, Poage GM, Koestler DC, Langevin SM, et al In utero exposures, infant growth, and DNA methylation of repetitive elements and developmentally related genes in human placenta. Environmental health perspectives. 2012;120(2):296–302. Epub 2011/10/19. doi: 10.1289/ehp.1103927 . 22005006 10.1289/ehp.1103927 PMC3279448

[24] Cai J, Zhao Y, Liu P, Xia B, Zhu Q, Wang X, et al Exposure to particulate air pollution during early pregnancy is associated with placental DNA methylation. Science of The Total Environment. 2017;607–608(Supplement C):1103–8. https://doi.org/10.1016/j.scitotenv.2017.07.029. 10.1016/j.scitotenv.2017.07.029 28724248

[25] Hermann A, Goyal R, Jeltsch A. The Dnmt1 DNA-(cytosine-C5)-methyltransferase methylates DNA processively with high preference for hemimethylated target sites. The Journal of biological chemistry. 2004;279(46):48350–9. Epub 2004/09/02. doi: 10.1074/jbc.M403427200 . 15339928 10.1074/jbc.M403427200

