# Supplementary material for: Air pollution during pregnancy and placental adaptation in the levels of global DNA methylation
Source: PLoS One. 2018 Jul 6;13(7):e0199772. doi: 10.1371/journal.pone.0199772 (PMC6034814; doi:10.1371/journal.pone.0199772)
Supplement: S1 Table — Based on PM concentration, there were not any significant correlation between birth outcome including gestational age, weight, length, and head and chest circumference at the time of birth and PM2.5 and PM10 concentrations in whole pregnancy or each trimester (p>0.05). (DOCX) [file pone.0199772.s001.docx]

S1 Table. Relationship between fine particulate matter and birth outcomes

|  |  | PM2.5.  Whole pregnancy | PM2.5.  Trimester.1 | PM2.5  Trimester.2 | PM2.5  Trimester.3 | PM10  Whole pregnancy | PM10  Trimester.1 | PM10  Trimester.2 | PM10  Trimester.3 |
| --- | --- | --- | --- | --- | --- | --- | --- | --- | --- |
| Birth age, week | Spearman's rho | .097 | .117 | -.031 | .087 | .108 | .110 | .031 | .193 |
|  | p-value | .379 | .285 | .775 | .428 | .325 | .318 | .776 | .077 |
| Birth weight, gr | Spearman's rho | -.036 | -.028 | -.118 | .031 | .097 | .117 | .081 | .037 |
|  | p-value | .744 | .799 | .290 | .781 | .381 | .292 | .467 | .737 |
| Birth length, cm | Spearman's rho | -.103 | .019 | -.207 | -.082 | -.022 | .103 | -.104 | .036 |
|  | p-value | .353 | .866 | .059 | .457 | .843 | .352 | .345 | .742 |
| Chest circumference, cm | Spearman's rho | .059 | -.039 | -.005 | .122 | .068 | .059 | .002 | .105 |
|  | p-value | .622 | .741 | .966 | .303 | .567 | .620 | .985 | .375 |
| Head circumference, cm | Spearman's rho | -.082 | -.113 | -.170 | -.033 | -.156 | .064 | -.161 | -.203 |
|  | p-value | .478 | .328 | .140 | .774 | .175 | .581 | .163 | .076 |

Based on PM concentration, there were not any significant correlation between birth outcome including gestational age, weight, length, and head and chest circumference at the time of birth and PM2.5 and PM10 concentrations in whole pregnancy or each trimester (p>0.05)
