# Supplementary material for: Air pollution during pregnancy and placental adaptation in the levels of global DNA methylation
Source: PLoS One. 2018 Jul 6;13(7):e0199772. doi: 10.1371/journal.pone.0199772 (PMC6034814; doi:10.1371/journal.pone.0199772)
Supplement: S2 Table — In all subjects, there were not any significant correlations between placental global DNA methylation levels with maternal and gestational ages, pre gestational BMI, gravity and parity (p-value>0.05). (DOCX) [file pone.0199772.s002.docx]

S2 Table. Relationship between Placental DNA methylation levels and modification factors

|  | **Placental Global DNA methylation levels** | |
| --- | --- | --- |
|  | Spearman's rho | p-value |
| weight Gain during pregnancy | 0.035 | 0.77 |
| Age | 0.005 | 0.96 |
| Pre. Gestational BMI, Kg/m^2^ | -0.05 | 0.67 |
| Gravity | 0.052 | 0.65 |
| Gestational Age | -0.097 | 0.40 |

In all subjects, there were not any significant correlations between placental global DNA methylation levels with maternal and gestational ages, pre gestational BMI, gravity and parity (p-value>0.05)
