# Supplementary material for: Air pollution during pregnancy and placental adaptation in the levels of global DNA methylation
Source: PLoS One. 2018 Jul 6;13(7):e0199772. doi: 10.1371/journal.pone.0199772 (PMC6034814; doi:10.1371/journal.pone.0199772)
Supplement: S3 Table — Regarding to birth outcomes, There were not any significant correlation between placental global DNA methylation and birth outcome including gestational age, weight, length, and head and chest circumference at the time of birth (p>0.05). (DOCX) [file pone.0199772.s003.docx]

S3 Table. Relationship between Placental DNA methylation levels and birth outcomes

|  | **Placental Global DNA methylation levels** | |
| --- | --- | --- |
|  | Spearman's rho | p-value |
| Birth weight, gr | -.035 | .037 |
| Birth length, cm | .090 | .095 |
| Chest circumference, cm | -.081 | .070 |
| Head circumference, cm | .023 | .034 |

Regarding to birth outcomes, There were not any significant correlation between placental global DNA methylation and birth outcome including gestational age, weight, length, and head and chest circumference at the time of birth (p>0.05).
